# Invasion of Ancestral Mammals into Dim-light Environments Inferred from Adaptive Evolution of the Phototransduction Genes

**DOI:** 10.1038/srep46542

**Published:** 2017-04-20

**Authors:** Yonghua Wu, Haifeng Wang, Elizabeth A. Hadly

**Affiliations:** 1 School of Life Sciences, Northeast Normal University, 5268 Renmin Street, Changchun, 130024, China; 2 Jilin Provincial Key Laboratory of Animal Resource Conservation and Utilization, Northeast Normal University, 2555 Jingyue Street, Changchun, 130117, China; 3 Department of Bioengineering, Stanford University, Stanford, California 94305, USA; 4 Department of Biology, Stanford University, 371 Serra Mall, Stanford, CA 94305-5020, USA

## Abstract

Nocturnality is a key evolutionary innovation of mammals that enables mammals to occupy relatively empty nocturnal niches. Invasion of ancestral mammals into nocturnality has long been inferred from the phylogenetic relationships of crown Mammalia, which is primarily nocturnal, and crown Reptilia, which is primarily diurnal, although molecular evidence for this is lacking. Here we used phylogenetic analyses of the vision genes involved in the phototransduction pathway to predict the diel activity patterns of ancestral mammals and reptiles. Our results demonstrated that the common ancestor of the extant Mammalia was dominated by positive selection for dim-light vision, supporting the predominate nocturnality of the ancestral mammals. Further analyses showed that the nocturnality of the ancestral mammals was probably derived from the predominate diurnality of the ancestral amniotes, which featured strong positive selection for bright-light vision. Like the ancestral amniotes, the common ancestor of the extant reptiles and various taxa in Squamata, one of the main competitors of the temporal niches of the ancestral mammals, were found to be predominate diurnality as well. Despite this relatively apparent temporal niche partitioning between ancestral mammals and the relevant reptiles, our results suggested partial overlap of their temporal niches during crepuscular periods.

Mammals are hypothesized to have experienced selection for nocturnality during their early evolution^1^,^2^,^3^,^4^ and some argue nocturnality emerged in the Late Carboniferous almost 300 mya^5^, well before the origin of Mammalia. The nocturnality of the ancestral mammals is primarily inferred from the enhanced hearing and olfaction based on morphologic traits of Mesozoic fossil mammals^6^,^7^ and reduced daytime vision and activity(e.g., dichromatic color vision) in most living mammals^8^. Phenotypic traits preserved in early mammalian fossils such as diminutive body size, a less sprawling body posture, secondary palate and turbinals, etc. are consistent with the evolution of endothermy, which would enable mammals to be active at night when temperatures are lower and are consistent with the nocturnality hypothesis^9^. Evolution of increasing nocturnality of ancestral mammals is hypothesized to be a result of competition and/or predation of reptiles, which are predominately diurnal due to their ectothermic dependence on external warmth to achieve optimum activity^1^,^3^,^10^,^11^. While these studies have advanced our understanding of the diel activity pattern of ancestral mammals, a robust test for the invasion of ancestral mammals into the nocturnal niche using molecular data that specifically targets visual ability has not been conducted in a phylogenetic framework.

In vision, phototransduction is a process involving a signal transduction cascade that converts light into an electrical signal in photoreceptor cells. In the vertebrate retina two types of photoreceptor cells,(i.e., rods and cones) are specialized for different aspects of vision. Rods are extremely sensitive to light and are specialized for dim-light vision(e.g., scotopic vision), while cones have high spatial resolution and are dedicated to bright-light vision(e.g., photopic vision). The functional specializations of rods and cones are a result of different genes involved in their phototransduction cascades^12^. However, despite using different genes, the phototransduction cascades in rods and cones are similar and both start with the absorption of light in visual pigments that activates the opsins. The opsins then activate transducin(Gt), which subsequently activates the cGMP phosphodiesterase(PDE), reducing concentration of cGMP and eventually leading to closure of a cyclic nucleotide-gated(CNG) ion channel and hyperpolarizing the cell(Fig. 1). Along with the activation of these phototransduction cascades, a timely photoresponse recovery is responsible for a high temporal resolution, which is considered to be important for motion detection^13^.

The differentiation of the dim-light vision genes and the bright-light vision genes in photoreceptors provides an opportunity to test the nocturnality hypothesis of ancestral mammals. We would expect that a switch from diurnality to nocturnality in ancestral mammals would be accompanied by a strong positive selection for dim-light vision and that this selection would not have occurred in other diurnal tetrapod clades. In support of this, one recent study in raptors reveals that the phototransduction genes evolution is consistent with their diel activity variations, showing that the diurnal raptors(Accipitriformes) feature a positive selection for bright-light vision, while the nocturnal owls feature a positive selection for dim-light vision, suggesting a molecular phyloecological approach to investigate different diel activity patterns through analyzing the adaptive evolution of the bright-light vision genes and the dim-light vision genes^14^. In this study, with phototransduction gene sequences available from a broad range of tetrapods(Supplementary Table 1), we analyzed the adaptive evolution of 34 phototransduction-related genes that are known to be involved in rod and cone phototransduction pathway^12^(Fig. 1) within a phylogenetic context(Fig. 2). Our results indicate increasing selection for nighttime vision within mammals, consistent with the primarily nocturnal lifestyles of extant taxa. Consistent with the recent study in birds^14^, we find concordant patterns between the diel activity patterns and the selection of the phototransduction genes within both mammals and reptiles. Our study highlights the power of using phototransduction genes to predict and/or reconstruct the diel activity patterns of both extant and extinct taxa using a phylogenetic context.

## Materials and Methods

### Data collection

The coding sequences of 34 vision genes involved in the phototransduction pathway were downloaded from the public databases GenBank and Ensembl(Supplementary Table 1). To maximize phylogenetic coverage, one or two represented species(depending on their sequences availabilities) from each major taxa of amniotes were used. The sequences of each gene were aligned using software webPRANK(http://www.ebi.ac.uk/goldman-srv/webprank/)^15^. The algorithm implemented in this software is considered to generate a more reliable alignment than other alignment methods and is capable of reducing false positive results of positive selection inference based on the branch-site model^16^. During the analyses, the sequence alignments were manually inspected for quality and the species with short sequences or low sequence identities(e.g., long indels and multiple ambiguous bases Ns) were removed. After such pruning, only the high-quality alignment results were used for subsequent positive selection analyses.

### Positive selection analyses

We used codon-based maximum likelihood methods implemented in the Codeml program in PAML 4.8a package^17^ for our positive selection analyses. The maximum likelihood methods estimate the ratio of non-synonymous to synonymous substitutions per site(d_N_/d_S_ or ω), and *ω* < 1, *ω* = 1 and *ω* > 1 indicate purifying selection, neutral evolution and positive selection, respectively. For our analyses, the species relationships(Fig. 2) were constructed based on published studies^18^,^19^,^20^,^21^,^22^,^23^, and the branch model, branch-site model and clade model C were used to detect positive selection along the branches or clades of interest. For the analyses, each branch or clade of interest was respectively used as the foreground branch or clade while all others were treated as the background. Likelihood ratio tests(LRT) were then employed to compare the goodness of fit of the more complex models with their corresponding null models.

We first used the two-rate branch model to test the strength and nature of selection along the foreground branches of interest. The two-rate model allows ω variations between foreground branches and background branches, and its goodness of fit was compared with the one-rate model, which assumes one single ω value for every branches base on the LRT. To further examine if the ω value of the foreground branch is greater than one, the likelihood values of the two-ratio model was then compared with the two-ratio model with a constraint of ω = 1.

We also used a more powerful branch-site model to detect positively selected sites along the branches of interest. With this analyses, we used the recommended Test 2, which compares a modified model A with its corresponding null model with ω = 1 fixed. The modified model A assumes four classes of sites. The site class 0(0 < ω_0_ < 1) and site class 1(ω_1_ = 1) respectively include evolutionarily conserved and neutral codons in both background branches and foreground branches, while the site classes 2a and 2b respectively include evolutionarily conserved(0 < ω_0_ < 1) or neutral(ω_1_ = 1) codons in the background branches, but are allowed to be under positive selection(ω_2_ > 1) along the foreground branches. When the LRT is significant, the Bayes Empirical Bayes method was used to identify positively selected sites.

We used the clade model C to identify the sites under divergent selection pressure between the foreground clade and the background clade. Clade model C assumes three classes of sites and the site class 0(0 < ω_0_ < 1) and site class 1(ω_1_ = 1) includes evolutionarily conserved and neutral codons, respectively, while the site classes 2 allows codons under divergent selection pressures between foreground clade and background clade. The clade model C was compared with a modified null model M2a_rel for LRT since M2a_rel is believed to better account for among-site variation in selective constraint and help to reduce false positive results^24^.

### Robustness tests of positive selection genes

We examined the effects of the phylogenetic uncertainty and the initial value variations of kappa and omega on our positive selection results. To examine the effects of phylogenetic uncertainty on our results, we used another mammalian phylogeny that differs from the molecular phylogeny we used in several aspects. The new mammalian phylogeny is based on both molecular and morphological data^25^ and one of its main differences from our phylogeny is the separation of Xenarthra from Afrotheria, and Xenarthra is treated as a sister taxa of all other placentals. In addition, several other inconsistences within Afrotheria and Euarchonta are also noted, for instance, Scandentia is treated as more closely related to Dermoptera than to Glires. In addition to the phylogenetic uncertainty, we also evaluated the dependences of our positive selection results on the initial value variations of kappa and omega. For this, we respectively used two different initial values of kappa(kappa = 0.5, 3.0) and omega(omega = 0.5, 2.0) for our positive selection analyses.

### Ancestral sequence reconstruction

We reconstructed ancestral amino acid sequences of *LWS, RH1, SWS1* and *SWS2* to analyze their possible amino acid replacements along branches of interests. For this, we used the amino acid-based marginal reconstruction implemented in the empirical Bayes approach in PAML 4.8a^26^. With the marginal reconstruction, the character was assigned to one single interior node and the character with the highest posterior probability is used as the best reconstruction. Two different amino acid substitution models, JTT and Poisson, were used to examine the consistency of our results. The model JTT assumes different substitution rates of different amino acids while the Poisson model assumes the same substitution rate of all amino acids. The analyses based on the JTT and Poisson models generated similar results, and for convenience only the results based on the JTT model are shown.

### Protein structure reconstruction

To understand the potential effects of the positively selected site and the critical amino acid replacement S159A of *LWS* in the ancestral mammals, we reconstructed three-dimensional structure of *LWS* at the internal node representing the last common ancestor of modern mammals based on the ancestral amino acid reconstruction mentioned above. For 3D structure reconstruction, we used homology modelling techniques implemented in SWISS-MODEL(http://swissmodel.expasy.org/)^27^. The 3D structure was reconstructed based on the template 2j4y.1.A, which is the first best hit according to the global quality estimation(GMQE).

### Analyses of gene losses in reptiles

We failed to find sequences of the two dim-light vision genes *PDE6A* and *GNGT1* from all reptiles(including birds) in nr/nt database, suggesting their losses from all reptiles studied. To further test the possible losses of these two genes in reptiles, we respectively used coding sequences of these two genes from primitive mammals(platypus and Tasmanian devil) as a query to blast against the database of whole genome-shotgun contigs(WGS) of reptiles in GenBank, and no sequences for *PDE6A* or *GNGT1* were found in reptiles. Specifically, for *PDE6A*, we used both its whole coding sequences and each of its six longest exon sequences of playtupus(*Ornithorhynchus anatinus*) as queries to blast against WGS of reptiles using web-based blastn. Similarly, for *GNGT1*, we used both its whole coding sequences and each of its two exon sequences of Tasmanian devil(*Sarcophilus harrisii*) to blast against WGS of reptiles. The best hits(e < = 1e - 5) of *PDE6A* and *GNGT1* were subsequently used to blast against nr/nt database using the web-based blastn and almost all the best hits showed 100% coverage and 100% sequence identity with genes *PDE6B*/ *PDE6C* and *GNGT2*/*GNG11*, respectively, instead of our target *PDE6A* and *GNGT1*, suggesting the losses of the two dim-light vision genes from all the reptiles studied.

## Results and Discussion

### Evolution of nocturnality in mammals

**Figure 1:**
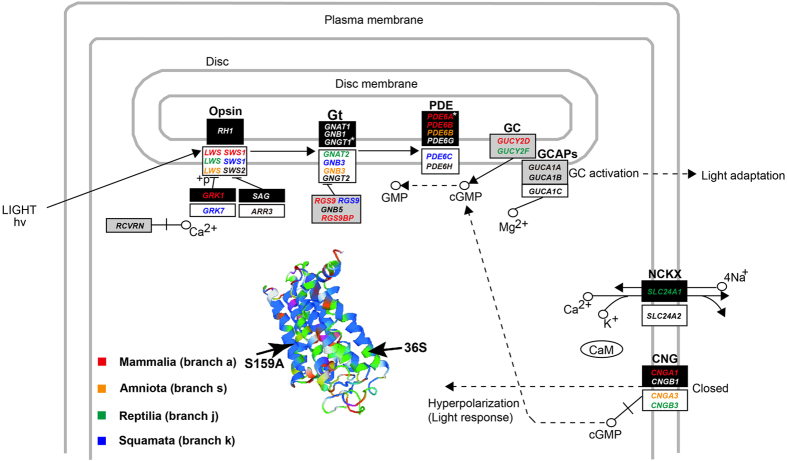
Positively selected genes involved in the phototransduction pathway in rods(according to KEGG pathway: map04744). For convenience, the genes involved in the phototransduction pathway in cones are also shown. Dark rectangles, white rectangles and grey rectangles show genes involved in the phototransduction pathway of rods, cones and both, respectively^12^. Positively selected genes found along the branches of Mammalia, Amniota, Reptilia and Squamata(Fig. 2) are shown in red, yellow, green and blue, respectively. *Shows sequences of two dim-light vision genes *GNGT1* and *PDE6A* are unavailable from all reptiles studied. See text for the meanings of protein abbreviations and the functions of their corresponding genes. Solid line shows direct interaction and dashed line shows indirect interaction. The three-dimensional structure of the long-wavelength sensitive opsin *LWS* of the ancestral mammals is also shown. S159A represents the amino acid replacement associated with the spectral turning of *LWS*, and 36 S indicates the positively selected site with a high posterior probability(>0.95).

**Figure 2:**
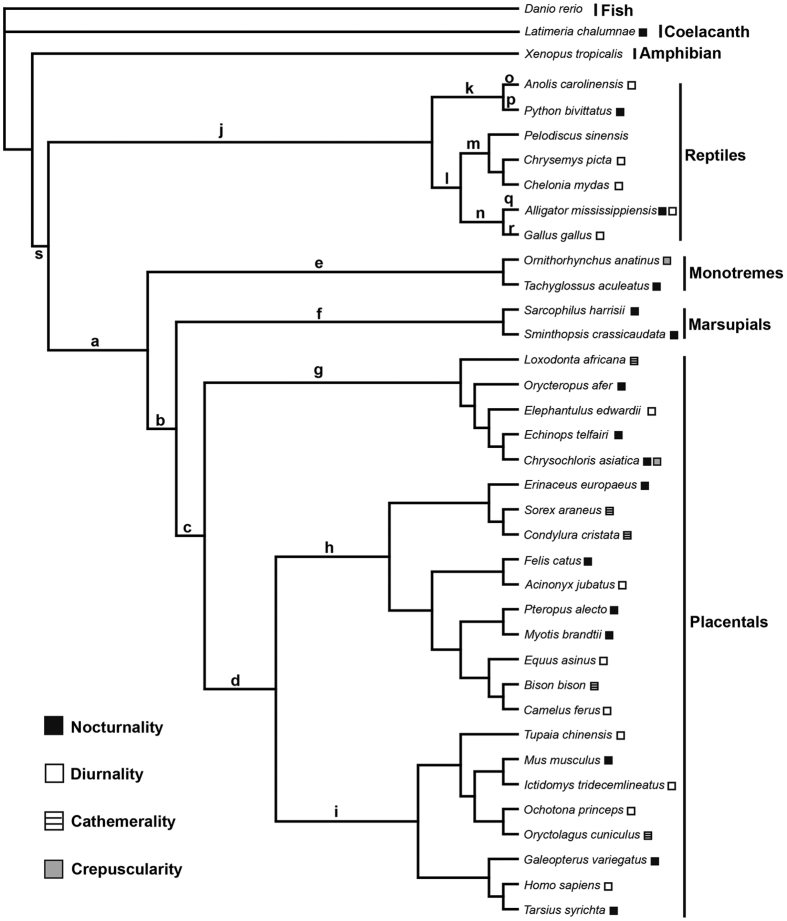
The species tree of the represented species used in this study. The phylogenetic relationships among species follow previous studies^18^,^19^,^20^,^21^,^22^,^23^. The letters a-s show branches used in positive selection analyses. Noting that species used for different genes analyses are different due to their sequence availabilities. The diel activity patterns of species are also shown. Please refer to Supplementary Table 1 for details.

We tested for adaptive evolution of vision genes using different models implemented in PAML^17^. Positively selected genes(PSGs) were only identified when using the branch-site model(Table 1, Supplementary Table 2), while no PSGs were detected with the branch model and the clade model C. The variable capabilities of these models in identifying PSGs may be partly attributed to their different underlying assumptions. For example, it has been shown that positive selection is less likely to affect all sites, and thus the ω values averaged over all sites by the branch model is almost never >1^28^. Similarly, for the clade model C, the ω values are calculated as an average over all lineages in certain clades^29^. The ω values averaged over all sites or all lineages may have weakened power in detecting positive selection. On the other hand, the branch-site model is built on an underlying hypothesis that positive selection tends to affect only a few sites along particular lineages^28^, which may explain why the branch-site model has improved power in detecting PSGs compared with other models. For the PSGs identified by the branch-site model, their positive selection signals remained robust when phylogenetic uncertainty and variations of the initial values of kappa and omega(ω) in the positive selection analyses were considered. Specifically, to test the possible occurrence of nocturnality in ancestral mammals, we analyzed the adaptive evolution of vision genes along the ancestral branch(branch a in Fig. 2) of all three extant clades of mammals(Monotremata, Marsupialia and Placentalia) and we found four dim-light vision genes(*GRK1, PDE6A, PDE6B* and *CNGA1*) under positive selection(Fig. 1, Table 1). Of the four PSGs, *PDE6A, PDE6B* and *CNGA1* are involved in the activation of phototransduction pathway, and *GRK1* is involved in the photoresponse recovery. *PDE6A* and *PDE6B* are known to encode two hydrolytic subunits *α* and *β* of phosphodiesterase and *CNGA1* encodes a protein forming CNG ion channel. *GRK1* plays a role in deactivation of activated rhodopsin(*RH1*) and contributes to rhodopsin recovery. Additionally, two other vision genes(*GUCY2D* and *RGS9*) involved in photoresponse recovery were found to be under positive selection. *GUCY2D* encodes guanylyl cyclase involved in the resynthesis of cGMP, and *RGS9* plays a role in deactivation of transducin. Moreover, *RGS9BP*, which plays a role in inactivation of transducin, was found to be under positive selection along the branch(combined branches a and b due to sequence unavailability of monotremes) leading to Theria. These PSGs involved in both the phototransduction activation and the photoresponse recovery likely contribute to the evolution of increased visual sensitivity as well as enhanced motion detection capability in ancestral mammals in dim-light conditions.

We also found the long-wavelength sensitive opsin *LWS* and the short-wavelength sensitive opsin *SWS1* were under positive selection in ancestral mammals(Fig. 1, Table 1). *LWS* showed strong positive selection along the common ancestor branch(branch a) of extant mammals, and *SWS1*, which is lost from monotremes^1^, showed positive selection along the branch(combined branches a and b) leading to Theria. Regarding the strong positive selection of *LWS*, we further used the Bayes Empirical Bayes model^30^ to identify the positively selected sites and only one such site was detected with a high posterior probability(0.956), which was determined to be located in the transmembrane domain(Fig. 1, Supplementary Fig. 1). We subsequently reconstructed the ancestral sequence of *LWS* based on an empirical Bayes approach^26^ and 19 amino acid replacements along the ancestral mammal branch were found(Supplementary Fig. 2). Among the 19 amino acid replacements, one critical amino acid replacement(S159A, corresponding to S164A in bovine rhodopsin) that is known to decrease the wavelength of maximal absorption(λmax) of *LWS* by 7 nm was identified^31^(Supplementary Fig. 2, Supplementary Table 3), suggesting a short-wavelength shift of *LWS*. Given the short-wavelength shift of *LWS*, we further reconstructed ancestral sequences of three other opsins *SWS1, SWS2* and *RH1* along the ancestral mammalian branch; we did not find any critical amino acid replacements associated with their spectral turnings(Supplementary Table 3)(data not shown), suggesting their λmax values were unchanged.

The short-wavelength shift of *LWS* may have helped ancestral mammals to maximize photon absorption by tuning their *LWS* spectral sensitivity to the relatively abundant short-wavelength light in twilight^32^. If this is the case, our results suggest crepuscular activity of ancestral mammals along with increasing nocturnality, consistent with a recent study that argues a ‘twilight phase’ of mammalian evolution given the possible retention of three cone opsins(*LWS, SWS1* and *SWS2*)^33^. Moreover, the positive selection results found in ancestral mammals are very similar with ancestral owls that also show the positive selection of the dim-light vision genes(*SLC24A1, CNGB1*) and the cone opsin genes(*LWS* and *SWS2*), and particularly, the *LWS* of ancestral owls is found to harbor the same one critical amino acid replacement(corresponding to S164A in bovine rhodopsin) as that of ancestral mammals leading to a short-wavelength shift by reducing its λmax of 7 nm^14^. The similar results between ancestral mammals and ancestral owls may be a result of their adaption to similar diel activity patterns.

We further analyzed the selection on vision genes along the main branches within mammals(branches b-i in Fig. 2, Supplementary Table 2) to examine the additional adaptations to light environments. For the Monotremata branch(branch e), only *RGS9* was under positive selection, suggesting that there has been few vision modification along this branch since its evolution. Within Theria, sister to Monotremata, three PSGs(*GUCY2D, RGS9* and *RH1*) with strong positive selection signals were found along the ancestral therian branch(branch b). *GUCY2D* and *RGS9* are involved in photoresponse recovery. *RH1* is the rod-specific rhodopsin and further ancestral sequence reconstruction identified one critical amino acid replacement(N83D) leading to an increase of the λmax of *RH1* by 2 nm(Supplementary Table 3) along the ancestral therian branch(Supplementary Fig. 3). This long-wavelength shift of *RH1* may assist in maximizing photon absorption in nocturnal conditions when relatively long-wavelength light dominates^34^. The strong positive selection of these three genes trends toward further enhancement of both the dim-light vision and the photoresponse recovery in the ancestral therians subsequent to stem mammals. Moreover, testing for positive selection within Theria showed that ancestral Marsupialia(branch f) harbored two PSGs including one dim-light vision gene(*RH1*) and one bright-light vision gene(*CNGA3*). Within ancestral Placentalia(branch c or combined branches b and c) one dim-light vision gene(*PDE6B*) and one photoresponse recovery gene(*GUCA1B*) were found to be under positive selection. Our results support a trend of increased adaptation to the dim-light conditions from ancestral Mammalian through Theria and to ancestral Placentalia. A further examination of positive selection along the main branches(branches g-i) within Placentalia only found two PSGs(*RH1* and *SWS1*) along the ancestral branch(branch h) of Laurasiatheria, while no PSGs were detected along the other two branches(branches g and i) including Euarchontoglires, Afrotheria and Xenarthra, suggesting fewer subsequent modifications in vision in these groups. Thus these three subclades(Euarchontoglires, Afrotheria and Xenarthra) might retain pleisiomorphic visual adaptation similar to ancestral placentals, perhaps in part be due to their relatively early origins prior to the K/T boundary^22^,^35^, a time when diurnal niches were occupied by other species and dinosaurs still dominated terrestrial habitats. Considering our partial taxa coverage for each of the three subclades, further studies incorporating more samples would be useful for a robust inference of their diel activity patterns.

### Nocturnality as a derived trait

To further test whether the possible nocturnality of ancestral mammals evolved from a diurnal ancestor, we examined the adaptive evolution of vision genes along the common ancestral branch of amniotes(branch s in Fig. 2). Among the 34 genes analyzed, 4 genes(*CNGA3, GNB3, LWS* and *PDE6B*) which are involved in the activation of the phototransduction pathway were found to be under positive selection(Fig. 1, Table 1). Among the four PSGs, *CNGA3, GNB3* and *LWS* are bright-light vision genes and *PDE6B* is a dim-light vision gene. *CNGA3*, which encodes a subunit of the CNG ion channel, presented the strongest positive selection signal(ω_2a_ = 447.444, ω_2b_ = 447.444, df = 1, *P* = 0.002). *GNB3* encodes the *β* subunit of transducin and showed the second strongest positive selection. *LWS* was under relatively strong positive selection and one positively selected site located in the transmembrane domain was identified(Supplementary Fig. 1). The only dim-light vision gene, *PDE6B*, showed the weakest positive selection signal compared with the other three bright-light vision genes. The relatively strong positive selection of the bright-light vision genes in ancestral amniotes probably helped to promote their visual acuity in daytime and suggests their predominately diurnal lifestyle. The adaptation of ancestral amniotes to bright-light environments may have occurred during the habitat shift from aquatic or semi-aquatic habitats of amphibian ancestors to their terrestrial habitats, where light is more intense and less attenuated by water.

Given the diurnality of early amniotes inferred here, the nocturnality of ancestral mammals is likely a derived character. Moreover, our finding of a few positively selected bright-light vision genes along the branch leading to extant mammals suggests that origin of dim-light vision and true nocturnality of the common ancestors of extant mammals likely occurred only once, while the timing of the occurrence of the nocturnality remains unknown. It is well known that mammals, as one of the members of synapsid lineage, appear relatively late and before their appearance, synapsids had experienced a long evolutionary history, which is sufficient to allow their adaptation to different niches(e.g., temporal niches)^5^,^7^. A recent phenotypic study implies that the invasion of synapsids into nocturnality may have occurred early extending back to the Late Carboniferous^5^. If this is the case, one possibility is that the nocturnality of ancestral mammals might be retained from that of their synapsid progenitors. However, uncertainties of the timing exist since the fossil synapsid taxa used in the phenotypic study may not represent the common ancestor of extant mammals, and hence the timing of the nocturnal invasion of the common ancestor of extant mammals still remains unknown.

### Temporal niches differentiation between ancestral mammals and reptiles

Invasion of ancestral mammals into nocturnality is hypothesized to be a result of competitive exclusion and/or predation by reptiles, which are believed to be predominately diurnal due to their reliance on solar radiation for warmth^36^. Among reptiles, the insectivorous lizards, like early mammals, occupy the insectivoran niche and may be one of the main competitors of ancestral mammals^10^,^36^. Here we tested the diurnality of reptiles by analyzing positive selection along different branches within reptiles(Fig. 2). The initial examination along the ancestral branch(branch j) of the extant reptiles studied showed very strong positive selection signals in five genes(Fig. 1, Table 1). Among the five PSGs, three genes *GNAT2, LWS* and *CNGB3* are bright-light vision genes and one gene *SLC24A1* is a dim-light vision gene. *GNAT2*, which encodes transducin *α* subunit, and *LWS* both showed the strongest positive selection signals. *CNGB3* is involved in the formation of CNG ion channel. The only positively selected dim-light gene *SLC24A1* showed relatively strong positive selection. *SLC24A1* encodes the Na^+^/Ca^2+^-K^+^ exchanger(NCKX), which removes free calcium in the rod outer segment and contributes to the restoration of the cGMP concentration and hence photoresponse recovery. In addition, *GUCY2F*, which encodes guanylyl cyclase involved in the resynthesis of cGMP and contributes to the photoresponse recovery, was also found to be under positive selection. Our results therefore suggest that positive selection for bright-light vision dominated the ancestral reptiles, and hence support the hypothesis that ancestral reptiles were predominately diurnal. In support of this, we failed to find two dim-light vision genes *PDE6A* and *GNGT1* from all reptiles(including birds) in both nr/nt database and the whole-genome shotgun contigs database, suggesting the loss of these genes in ancestral reptiles. Previous studies also find the absence of the *PDE6A* gene in birds and non-avian reptiles^37^,^38^. Additionally, no transcripts of *PDE6A* and *GNGT1* were detected in a recent study about the retinal transcriptome sequencing of 15 bird species including both diurnal and nocturnal taxa as well^14^. The possible loss of the two dim-light vision genes from ancestral reptiles may be due to relaxed selection on dim-light vision associated with their predominate diurnality. In addition to the ancestral reptiles, we detected almost only bright-vision genes to be under positive selection along the branches(branches k, o and p) leading to Squamata(Table 1, Supplementary Table 2), suggesting their predominate diurnality as well. We should note here that not all reptilian taxa we examined showed predominately diurnal activities, with the various branches related to turtles, crocodiles and birds harboring considerable PSGs related to the dim-light vision(Supplementary Table 2), demonstrating the abilities of their vision in dim-light.

### Methodological considerations

Here we detected the diel activity patterns of relevant taxa based on the molecular phyloecological approach. These patterns are largely consistent with traditional hypothesis that ancestral mammals are predominately nocturnal and reptiles are mainly diurnal^1^. This molecular phyloecological approach can be useful to reconstruct the ancestral character states of extinct taxa. However, we would like to note some limitations of this molecular phyloecological approach: i) The approach is only restricted to those taxa from which their gene sequences are available, and thus can not be applied to those extinct taxa lacking sequence availabilities or representatives of their derived living taxa. ii) Little is known about the effects of the absence of extinct taxa on the phylogenetic analyses of positive selection. Regarding our focal amniotes, there are numerous extinct species, while our study is only capable of involving living amniotes. Given that previous morphological studies show an improved ancestral trait reconstruction by incorporating fossil information relative to those based on extant taxa alone^39^,^40^,^41^, future integrated analyses incorporating both molecular and morphological results would lead to more robust inferences of ancestral character states. iii) Even for the taxa with numerous derived living taxa, the effects of using relatively less represented entities on the positive selection results are less clear. Though our study covers a broad range of tetrapods, the sample size is relatively small for each of certain subclades, and our study is largely restricted to those taxa from which their genomes are available. Nonetheless, one previous study shows that the sample sizes seem to have little effects on positive selection results^42^, and thus our positive selection results may still be informative. In general, an increased taxa sampling is considered to improve the power to detect positive selection^43^, so future study incorporating more taxa with intensive sampling in a supertree context may be more powerful in detecting positive selection. iv) For the large-scale phylogenetic analyses spanning a long evolutionary history, like our tetrapod phylogeny, the gene sequences among taxa would be highly divergent, which may lead to substitution saturation and possible false positive results. However, the branch-site model test has been demonstrated to be robust against highly divergent sequences and the substitution saturation is found to have nearly no effects on the false positive results^44^, suggesting the robustness of our positive selection results.

## Conclusion

Ancestral mammals are inferred to be nocturnal based on multiple lines of morphological evidence in fossils^7^,^8^,^45^. In this study, we conducted comparative evolutionary analyses of genes involved in vision pathways and revealed positively selected genes involved in dim-light vision within ancestral Mammalia, supporting the nocturnality of early mammals. The positively selected vision genes of ancestral mammals incorporated almost all of the principle components of the phototransduction pathway involved in both phototransduction activation and photoresponse recovery(Fig. 1). These results suggest substantial modification of the visual system within subsequent branches of Mammalia toward crown Placentalia trending toward an increased visual sensitivity and an enhanced ability to detect motion. These abilities may be important for mammals to hunt flying insects in dim-light conditions^7^. Our results further show that the nocturnality of the common ancestors of extant mammals was clearly derived from the predominately diurnal ancestral amniotes, perhaps driven in part by competition exclusion of predominately diurnal reptiles. Despite the large differentiation of the temporal niches between ancestral mammals and reptiles, our study provides evidence of both the crepuscularity of ancestral mammals and the dim-light activities of some reptile taxa, and thus suggests their possible partial overlap during crepuscular periods. Consistent with a recent bird study^14^, our study shows that the phototransduction genes evolution corresponds very well with the diel activity variations in mammals and reptiles, thus further tests the effectiveness of the molecular phyloecological approach for inferring the diel activity patterns, which remains to be addressed in other systems. Finally, our study includes partial representative taxa of reptiles and mammals, future studies incorporating more taxa from both groups may provide more profound information.

## Additional Information

**How to cite this article**: Wu, Y. *et al*. Invasion of Ancestral Mammals into Dim-light Environments Inferred from Adaptive Evolution of the Phototransduction Genes. *Sci. Rep.* **7**, 46542; doi: 10.1038/srep46542(2017).

**Publisher's note:** Springer Nature remains neutral with regard to jurisdictional claims in published maps and institutional affiliations.

## Supplementary Material

Supplementary Figures and Tables

Supplementary Dataset 1

## Figures and Tables

**Table 1:** Positively selected genes identified based on the branch-site model along major branches.

Taxa /Genes	Parameter estimates	2∆L	df	*p*-value	Positively Selected sites
**Amniota(branch s)**					
*CNGA3*	*p*_*0*_ = 0.846 *p*_*1*_ = 0.141 *p*_*2a*_ = 0.011 *p*_*2b*_ = 0.002	9.89	1	0.002	344Q, 453S
	*ω*_*0*_ = 0.034 *ω*_*1*_ = 1.000 *ω*_*2a*_ = **447.444** *ω*_*2b*_ = **447.444**				
*GNB3*	*p*_*0*_ = 0.951 *p*_*1*_ = 0.025 *p*_*2a*_ = 0.024 *p*_*2b*_ = 0.0006	10.24	1	0.001	13G
	*ω*_*0*_ = 0.023 *ω*_*1*_ = 1.000 *ω*_*2a*_ = **72.257** *ω*_*2b*_ = **72.257**				
*LWS*	*p*_*0*_ = 0.888 *p*_*1*_ = 0.105 *p*_*2a*_ = 0.006 *p*_*2b*_ = 0.0007	5.04	1	0.025	163C
	*ω*_*0*_ = 0.045 *ω*_*1*_ = 1.000 *ω*_*2a*_ = **32.555** *ω*_*2b*_ = **32.555**				
*PDE6B*	*p*_*0*_ = 0.933 *p*_*1*_ = 0.061 *p*_*2a*_ = 0.006 *p*_*2b*_ = 0.0004	8.18	1	0.004	292S, 688C
	*ω*_*0*_ = 0.037 *ω*_*1*_ = 1.000 *ω*_*2a*_ = **25.825** *ω*_*2b*_ = **25.825**				
**Mammalia(branch a)**					
*GUCY2D*	*p*_*0*_ = 0.977 *p*_*1*_ = 0.007 *p*_*2a*_ = 0.016 *p*_*2b*_ = 0.0001	6.90	1	0.009	135T
	*ω*_*0*_ = 0.016 *ω*_*1*_ = 1.000 *ω*_*2a*_ = **999.000** *ω*_*2b*_ = **999.000**				
*RGS9*	*p*_*0*_ = 0.951 *p*_*1*_ = 0.043 *p*_*2a*_ = 0.006 *p*_*2b*_ = 0.0003	8.75	1	0.003	191Y
	*ω*_*0*_ = 0.065 *ω*_*1*_ = 1.000 *ω*_*2a*_ = **577.912** *ω*_*2b*_ = **577.912**				
*LWS*	*p*_*0*_ = 0.888 *p*_*1*_ = 0.104 *p*_*2a*_ = 0.008 *p*_*2b*_ = 0.0009	5.60	1	0.018	36T
	*ω*_*0*_ = 0.045 *ω*_*1*_ = 1.000 *ω*_*2a*_ = **524.057** *ω*_*2b*_ = **524.057**				
*PDE6A*	*p*_*0*_ = 0.901*p*_*1*_ = 0.075 *p*_*2a*_ = 0.023 *p*_*2b*_ = 0.002	9.17	1	0.002	250T, 256L
	*ω*_*0*_ = 0.039 *ω*_*1*_ = 1.000 *ω*_*2a*_ = **43.385** *ω*_*2b*_ = **43.385**				372K, 681C, 710L
SWS1*	*p*_*0*_ = 0.902 *p*_*1*_ = 0.087 *p*_*2a*_ = 0.009 *p*_*2b*_ = 0.0009	7.39	1	0.007	53K, 228S
	*ω*_*0*_ = 0.054 *ω*_*1*_ = 1.000 *ω*_*2a*_ = **35.628** *ω*_*2b*_ = **35.628**				
RGS9BP*	*p*_*0*_ = 0.933 *p*_*1*_ = 0.043 *p*_*2a*_ = 0.023 *p*_*2b*_ = 0.001	5.41	1	0.020	138N, 175S
	*ω*_*0*_ = 0.076 *ω*_*1*_ = 1.000 *ω*_*2a*_ = **20.439** *ω*_*2b*_ = **20.439**				
*CNGA1*	*p*_*0*_ = 0.874 *p*_*1*_ = 0.111 *p*_*2a*_ = 0.013 *p*_*2b*_ = 0.002	5.82	1	0.016	499A
	*ω*_*0*_ = 0.033 *ω*_*1*_ = 1.000 *ω*_*2a*_ = **20.335** *ω*_*2b*_ = **20.335**				
*PDE6B*	*p*_*0*_ = 0.916 *p*_*1*_ = 0.057 *p*_*2a*_ = 0.026 *p*_*2b*_ = 0.002	15.23	1	9.510E-05	25I, 332S, 604T
	*ω*_*0*_ = 0.037 *ω*_*1*_ = 1.000 *ω*_*2a*_ = **12.460** *ω*_*2b*_ = **12.460**				610K, 612I, 668E
*GRK1*	*p*_*0*_ = 0.838 *p*_*1*_ = 0.070 *p*_*2a*_ = 0.084 *p*_*2b*_ = 0.007	4.17	1	0.041	2L, 230T, 258S
	*ω*_*0*_ = 0.044 *ω*_*1*_ = 1.000 *ω*_*2a*_ = **5.574** *ω*_*2b*_ = **5.574**				332D, 339I, 359G
**Reptilia(branch j)**					
*GNAT2*	*p*_*0*_ = 0.918 *p*_*1*_ = 0.077 *p*_*2a*_ = 0.005 *p*_*2b*_ = 0.0004	6.61	1	0.010	119V
	*ω*_*0*_ = 0.023 *ω*_*1*_ = 1.000 *ω*_*2a*_ = **999.000** *ω*_*2b*_ = **999.000**				
*LWS*	*p*_*0*_ = 0.883 *p*_*1*_ = 0.105 *p*_*2a*_ = 0.011 *p*_*2b*_ = 0.001	12.70	1	3.658E-04	225Q
	*ω*_*0*_ = 0.045 *ω*_*1*_ = 1.000 *ω*_*2a*_ = **999.000** *ω*_*2b*_ = **999.000**				
*SLC24A1*	*p*_*0*_ = 0.825 *p*_*1*_ = 0.159 *p*_*2a*_ = 0.013 *p*_*2b*_ = 0.003	9.12	1	0.003	134I, 147T
	*ω*_*0*_ = 0.051 *ω*_*1*_ = 1.000 *ω*_*2a*_ = **998.997** *ω*_*2b*_ = **998.997**				
*GUCY2F*	*p*_*0*_ = 0.831 *p*_*1*_ = 0.158 *p*_*2a*_ = 0.010 *p*_*2b*_ = 0.002	5.77	1	0.016	
	*ω*_*0*_ = 0.078 *ω*_*1*_ = 1.000 *ω*_*2a*_ = **465.122** *ω*_*2b*_ = **465.122**				
*CNGB3*	*p*_*0*_ = 0.723 *p*_*1*_ = 0.271 *p*_*2a*_ = 0.004 *p*_*2b*_ = 0.002	5.74	1	0.017	
	*ω*_*0*_ = 0.096 *ω*_*1*_ = 1.000 *ω*_*2a*_ = **96.904** *ω*_*2b*_ = **96.904**				
**Squamata(branch k)**					
*PDE6C*	*p*_*0*_ = 0.898 *p*_*1*_ = 0.097 *p*_*2a*_ = 0.004 *p*_*2b*_ = 0.0004	4.43	1	0.035	231I
	*ω*_*0*_ = 0.032 *ω*_*1*_ = 1.000 *ω*_*2a*_ = **273.863** *ω*_*2b*_ = **273.863**				
*SWS1*	*p*_*0*_ = 0.906 *p*_*1*_ = 0.086 *p*_*2a*_ = 0.007 *p*_*2b*_ = 0.0007	4.61	1	0.032	197I
	*ω*_*0*_ = 0.055 *ω*_*1*_ = 1.000 *ω*_*2a*_ = **21.161** *ω*_*2b*_ = **21.161**				
*GRK7*	*p*_*0*_ = 0.841 *p*_*1*_ = 0.145 *p*_*2a*_ = 0.012 *p*_*2b*_ = 0.002	7.16	1	0.007	112H, 258D
	*ω*_*0*_ = 0.078 *ω*_*1*_ = 1.000 *ω*_*2a*_ = **18.864** *ω*_*2b*_ = **18.864**				
*RGS9*	*p*_*0*_ = 0.926 *p*_*1*_ = 0.044 *p*_*2a*_ = 0.029 *p*_*2b*_ = 0.001	7.93	1	0.005	183N, 242I
	*ω*_*0*_ = 0.064 *ω*_*1*_ = 1.000 *ω*_*2a*_ = **7.223** *ω*_*2b*_ = **7.223**				257M, 316T, 390R
*GNB3*	*p*_*0*_ = 0.917 *p*_*1*_ = 0.023 *p*_*2a*_ = 0.059 *p*_*2b*_ = 0.001	4.95	1	0.026	60M, 162A, 174V
	*ω*_*0*_ = 0.021 *ω*_*1*_ = 1.000 *ω*_*2a*_ = **3.970** *ω*_*2b*_ = **3.970**				

Please see Fig. 2 for different branches and their corresponding taxa. For convenience, only the ω values of the foreground branches are shown. The positively selected sites with a high posterior probability support(>0.900) are also shown.

2∆L: twice difference of likelihood values between the modified model A and the corresponding null model with the ω = 1 fixed in the foreground branches; df: degrees of freedom; proportion of sites and their corresponding ω values in four site classes(*p*_*0*_, *p*_*1*_, *p*_*2a*_ and *p*_*2b*_) of the branch-site model are shown.*Shows genes sequences unavailable in monotremes and only the combined branches a and b were analyzed.
