# Supplementary material for: Invasion of Ancestral Mammals into Dim-light Environments Inferred from Adaptive Evolution of the Phototransduction Genes
Source: Sci Rep. 2017 Apr 20;7:46542. doi: 10.1038/srep46542 (PMC5397851; doi:10.1038/srep46542)
Supplement: Supplementary Figures and Tables [file srep46542-s1.pdf]

Supplementary Information

Invasion of Ancestral Mammals into Dim-light Environments Inferred from Adaptive Evolution of the Phototransduction Genes

Yonghua Wu, Haifeng Wang, Elizabeth A. Hadly

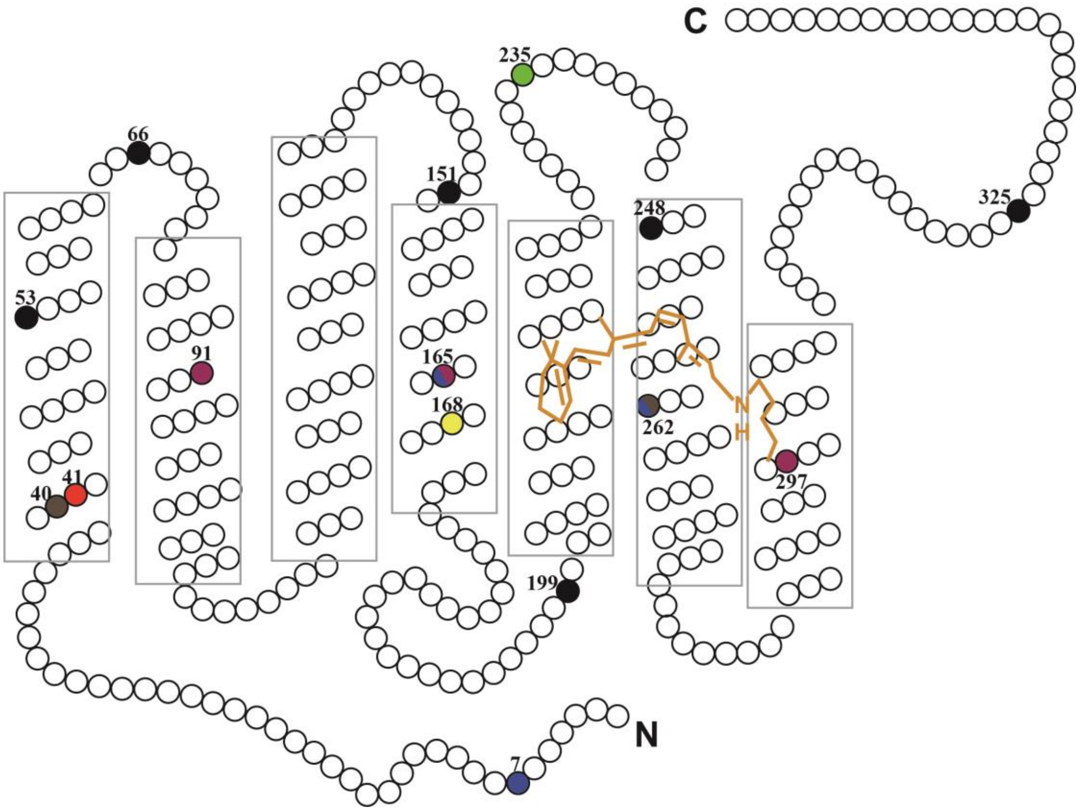

Supplementary Figure 1 Positively selected amino acid sites of *LWS* mapping on the secondary structure of bovine rhodopsin. The secondary structure is based on previous studies [31,46], and the 11-cis-retinal is shown in orange and seven transmembrane domains of rhodopsin are shown in grey rectangles. Only positively selected amino acid sites with a high posterior probability (>0.95) along different branches are shown in different colors. Mammals(red), reptiles(green), amniotes(yellow), snake(*Thamnophis sirtalis*)(blue), turtle(*Chrysemys picta*)(dark grey), crocodile(*Alligator mississippiensis*)(black) and bird(*Gallus gallus*)(violet).

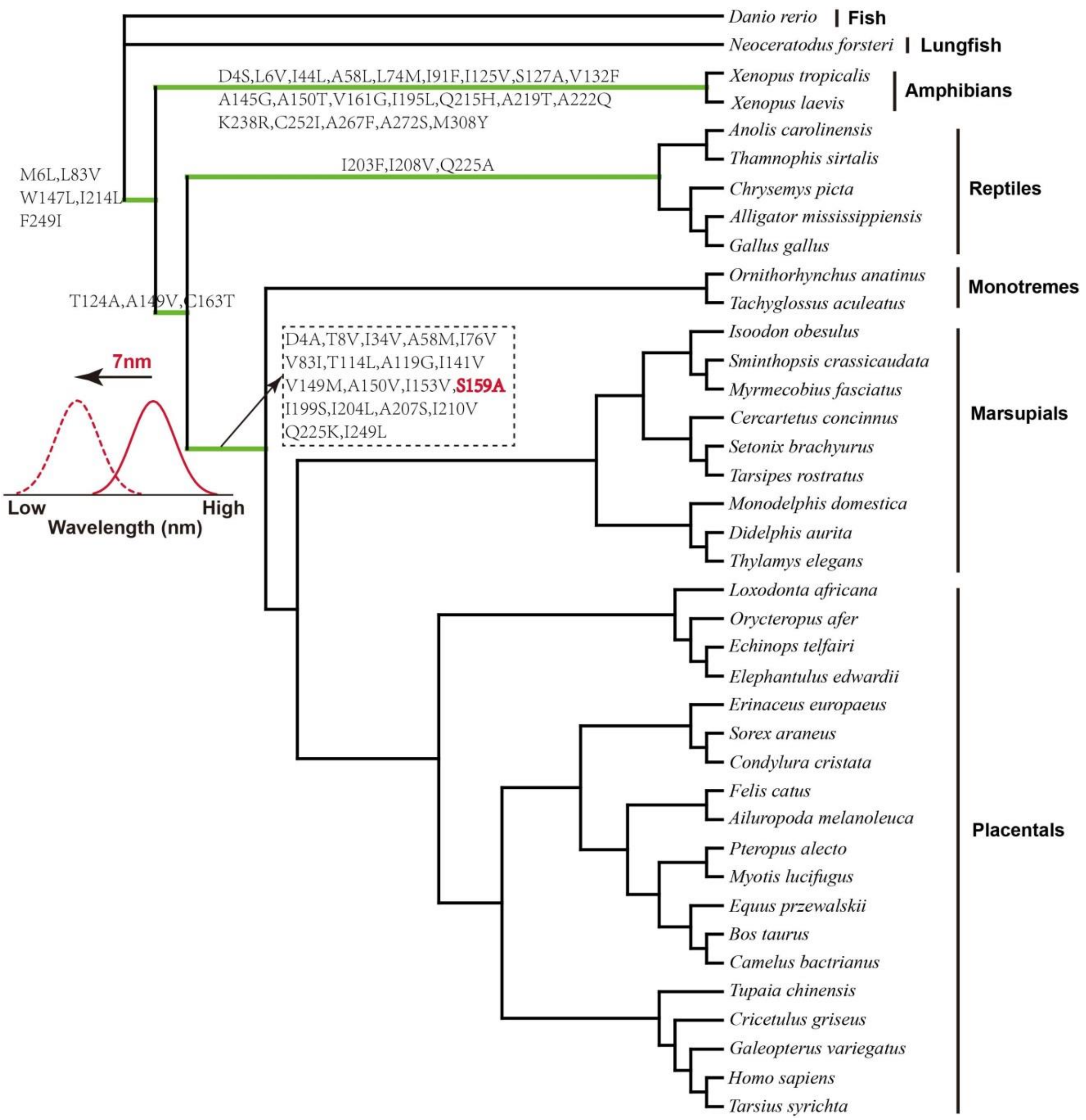

Supplementary Figure 2 The amino acids replacements of *LWS* along the branches of interest (green). The critical amino acid replacement (S159A) decreasing the λmax of *LWS* by 7nm along the ancestral mammalian branch is shown in red. The phylogenetic relationships among species follow previous studies [18-23]. The ancestral amino acid sequences of the internal nodes were reconstructed based on the empirical Bayes approach using the JTT model of the amino acid substitution.

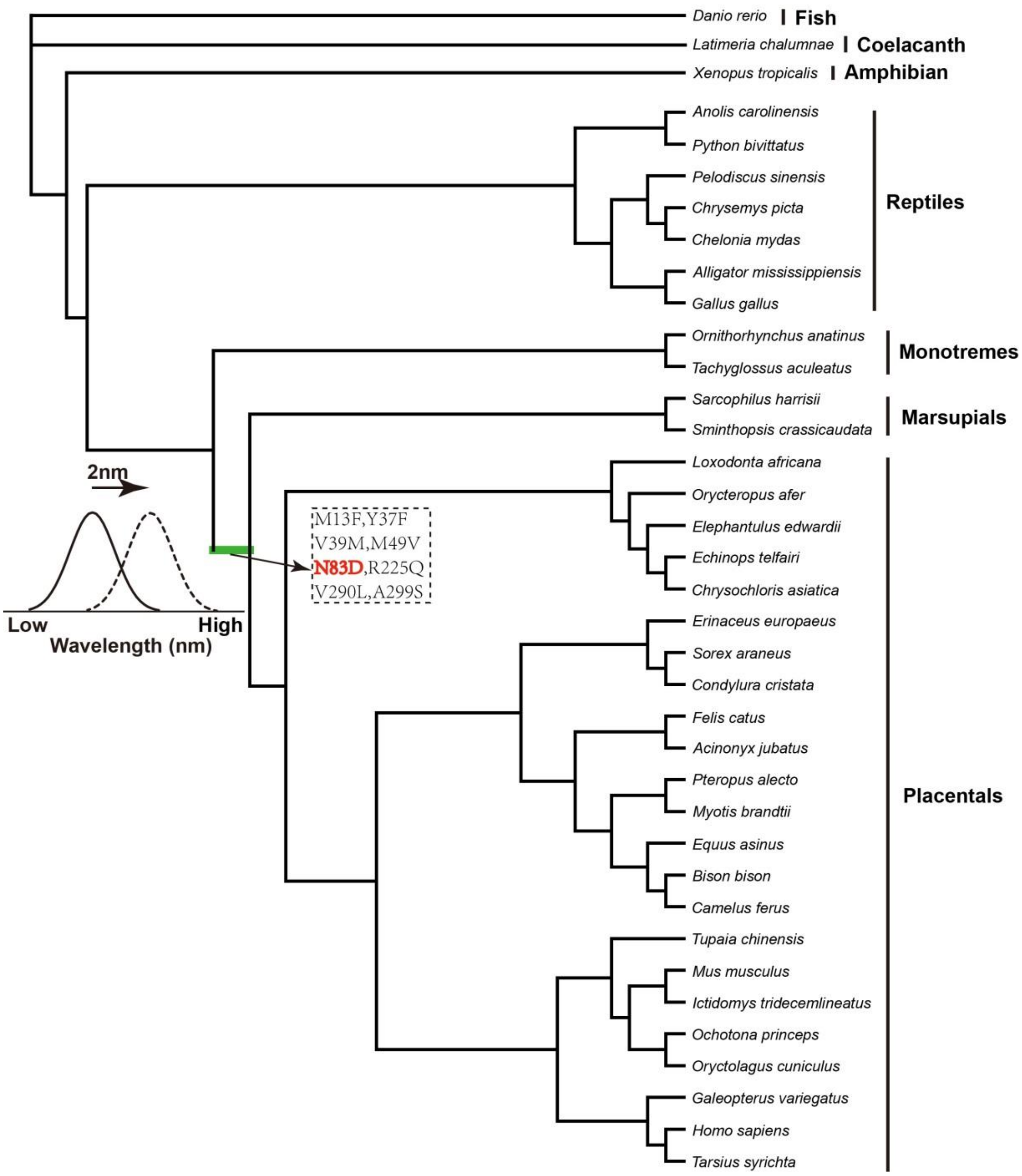

Supplementary Figure 3 The amino acids replacement of *RH1* along the common ancestor branch of Theria. The critical amino acid replacement (N83D) increasing the λmax of *RH1* by 2nm along the ancestral therian branch is shown in green. The phylogenetic relationships among species follow previous studies [18-23]. The ancestral amino acid sequences of the internal nodes were reconstructed based on the empirical Bayes approach using the JTT model of the amino acid substitution.

Supplementary Table 2 Positively selected genes identified based on the branch-site model along different branches of mammals and reptiles. Please see Fig.2 for different branches and their corresponding taxa. For convenience, only the ω values of the foreground branches are shown.

Taxa /Genes	Parameter estimates	2ΔL	df	*p*-value
**Theria (branch b)**				
GUCY2D	p_0=0.985 p_1=0.007 p_{2a}=0.007 p_{2b}=0.00005<br>ω_0=0.017 ω_1=1.000 **ω_{2a}=999.000 ω_{2b}=999.000**	3.99	1	0.046
RGS9	p_0=0.945 p_1=0.043 p_{2a}=0.012 p_{2b}= 0.0005<br>ω_0=0.065 ω_1=1.000 **ω_{2a}=998.999 ω_{2b}=998.999**	8.29	1	0.004
RH1	p_0=0.934 p_1=0.051 p_{2a}= 0.014 p_{2b}=0.0007<br>ω_0=0.040 ω_1=1.000 **ω_{2a}=148.023 ω_{2b}=148.023**	6.91	1	0.009
**Placentalia (branch c)**				
*GUCA1B**	p_0=0.885 p_1=0.085 p_{2a}=0.027 p_{2b}=0.003<br>ω_0=0.045 ω_1=1.000 **ω_{2a}=121.946 ω_{2b}=121.946**	4.04	1	0.044
PDE6B	p_0=0.936 p_1=0.061 p_{2a}=0.003 p_{2b}=0.0002<br>ω_0=0.038 ω_1=1.000 **ω_{2a}=77.324 ω_{2b}=77.324**	3.96	1	0.047
**Monotremata (branch e)**				
RGS9	p_0=0.948 p_1=0.045 p_{2a}=0.007 p_{2b}=0.0003<br>ω_0=0.064 ω_1=1.000 **ω_{2a}=288.444 ω_{2b}=288.444**	4.54	1	0.033
**Marsupialia (branch f)**				
CNGA3	p_0=0.850 p_1=0.144 p_{2a}=0.005 p_{2b}=0.0008<br>ω_0=0.034 ω_1=1.000 **ω_{2a}=21.009 ω_{2b}=21.009**	4.33	1	0.037
RH1	p_0=0.920 p_1=0.046 p_{2a}=0.032 p_{2b}=0.002<br>ω_0=0.040 ω_1=1.000 **ω_{2a}=22.100 ω_{2b}=22.100**	20.33	1	6.532E-06
**Laurasiatheria (branch h)**				
SWS1	p_0=0.903 p_1=0.087 p_{2a}=0.009 p_{2b}=0.0009<br>ω_0=0.054 ω_1=1.000 **ω_{2a}=130.950 ω_{2b}=130.950**	4.02	1	0.045
RH1	p_0=0.945 p_1=0.052 p_{2a}=0.003 p_{2b}=0.0002<br>ω_0=0.041 ω_1=1.000 **ω_{2a}=72.152 ω_{2b}=72.152**	6.72	1	0.009
**Lacertilia (branch o)**				
GRK1	p_0=0.889 p_1=0.071 p_{2a}=0.037 p_{2b}= 0.003<br>ω_0=0.046 ω_1=1.000 **ω_{2a}=242.953 ω_{2b}=242.953**	16.86	1	4.024E-05
**Serpentes (branch p)**				
GNGT2	p_0=0.755 p_1=0.191 p_{2a}=0.043 p_{2b}=0.011<br>ω_0=0.078 ω_1=1.000 **ω_{2a}=999.000 ω_{2b}=999.000**	4.80	1	0.028
LWS	p_0=0.880 p_1=0.104 p_{2a}=0.014 p_{2b}=0.002<br>ω_0=0.044 ω_1=1.000 **ω_{2a}=999.000 ω_{2b}=999.000**	11.27	1	7.866E-04
RCVRN	p_0=0.862 p_1=0.115 p_{2a}=0.021 p_{2b}=0.003<br>ω_0=0.049 ω_1=1.000 **ω_{2a}=998.989 ω_{2b}=998.989**	5.75	1	0.017
GNB3	p_0=0.962 p_1=0.025 p_{2a}=0.014 p_{2b}=0.0004<br>ω_0=0.023 ω_1=1.000 **ω_{2a}=348.239 ω_{2b}=348.239**	5.57	1	0.018
GNAT2	p_0=0.919 p_1=0.076 p_{2a}=0.004 p_{2b}=0.0003<br>ω_0=0.023 ω_1=1.000 **ω_{2a}=287.247 ω_{2b}=287.247**	4.22	1	0.040
**Archelosauria (branch l)**				
GRK1	p_0=0.895 p_1=0.074 p_{2a}=0.029 p_{2b}=0.002<br>ω_0=0.045 ω_1=1.000 **ω_{2a}=999.000 ω_{2b}=999.000**	11.27	1	7.875E-04
**Testudines (branch m)**				
GRK1	p_0=0.904 p_1=0.070 p_{2a}=0.025 p_{2b}=0.002	8.58	1	0.003

	ω_0=0.046 ω_1=1.000 **ω_{2a}=300.279 ω_{2b}=300.279**			
LWS	p_0=0.886 p_1=0.105 p_{2a}=0.008 p_{2b}=0.0009	3.93	1	0.048
	ω_0=0.044 ω_1=1.000 **ω_{2a}=11.596 ω_{2b}=11.596**			
**Archosauria (branch n)**				
GRK1	p_0=0.909 p_1=0.077 p_{2a}=0.013 p_{2b}=0.001	12.03	1	5.224E-04
	ω_0=0.046 ω_1=1.000 **ω_{2a}=285.763 ω_{2b}=285.763**			
PDE6B	p_0=0.900 p_1=0.057 p_{2a}=0.041 p_{2b}=0.003	7.43	1	0.006
	ω_0=0.037 ω_1=1.000 **ω_{2a}=59.329 ω_{2b}=59.329**			
**Crocodilia (branch q)**				
RCVRN	p_0=0.859 p_1=0.115 p_{2a}=0.023 p_{2b}=0.003	9.14	1	0.003
	ω_0=0.050 ω_1=1.000 **ω_{2a}=998.993 ω_{2b}=998.993**			
LWS	p_0=0.851 p_1=0.103 p_{2a}=0.041 p_{2b}=0.005	18.91	1	1.373E-05
	ω_0=0.044 ω_1=1.000 **ω_{2a}=959.005 ω_{2b}=959.005**			
GRK1	p_0=0.904 p_1=0.074 p_{2a}=0.020 p_{2b}=0.002	11.05	1	8.856E-04
	ω_0=0.046 ω_1=1.000 **ω_{2a}=56.257 ω_{2b}=56.257**			
PDE6B	p_0=0.878 p_1=0.056 p_{2a}= 0.062 p_{2b}=0.004	9.50	1	0.002
	ω_0=0.036 ω_1=1.000 **ω_{2a}=5.434ω_{2b}=5.434**			
**Neornithes (branch r)**				
SWS1	p_0=0.883 p_1=0.084 p_{2a}= 0.030 p_{2b}=0.003	6.33	1	0.012
	ω_0=0.054 ω_1=1.000 **ω_{2a}=668.008 ω_{2b}=668.008**			
SLC24A1	p_0=0.825 p_1=0.155 p_{2a}=0.017 p_{2b}=0.003	11.18	1	8.260E-04
	ω_0=0.051 ω_1=1.000 **ω_{2a}=393.261 ω_{2b}=393.261**			
GRK1	p_0=0.837 p_1=0.071 p_{2a}=0.085 p_{2b}=0.007	16.32	1	5.353E-05
	ω_0=0.046 ω_1=1.000 **ω_{2a}=230.465 ω_{2b}=230.465**			
SWS2	p_0=0.857 p_1=0.114 p_{2a}=0.026 p_{2b}=0.003	6.02	1	0.014
	ω_0=0.057 ω_1=1.000 **ω_{2a}=42.373 ω_{2b}=42.373**			
LWS	p_0=0.885 p_1=0.105 p_{2a}=0.009 p_{2b}=0.001	8.79	1	0.003
	ω_0=0.044 ω_1=1.000 **ω_{2a}=15.892 ω_{2b}=15.892**			

2ΔL: twice difference of likelihood values between two nested models; df: degrees of freedom; Proportion of sites and their corresponding ω values in four site classes (p_0, p_1, p_{2a} and p_{2b}) of the branch-site model are shown.* shows genes sequences unavailable in marsupials and only the combined branches b and c were analyzed.

Supplementary Table 3 Amino acid replacements of visual pigments (*LWS, SWS2, SWS1* and *RH1*) and their effects on the wavelength shift of maximal absorption (Δλ). Amino acid site numbers are based on the bovine rhodopsin.

Opsin	Amino acid replacement	Δλ (nm)	Reference
LWS			
	S164A	-7	[31]
	A164S	+6	[47]
	H181Y	-28	[31]
	Y261F	-8	[31]
	F261Y	+6	[47]
	T269A	-15	[31]
	A269T	+10	[47]
	A292S	-27	[31]
	S292A	+28	[47]
	S164A & H181Y	+11	[31]
SWS2			
	S91P	+10	[48]
	T93L	-9	[48]
	T93V	-6	[48]
	A94S	+14	[48]
	S127C	+2	[48]
	L207I	-6	[48]
	C211S	+2	[48]
	F261Y	+5	[48]
	A269S	+3	[48]
	A269T	+5	[48]
	S292A	+8	[48]
SWS1			
	F86Y	+66	[47]
	Y86F	-75	[47]

	F86S	+17	[47]
	S86F	-52	[47]
	S90G	-7	[47]
	S90C	-7	[47]
	C90S	+38	[47]
	I93T	-6	[47]
	E113D	-4	[47]
	D113E	-12	[47]
	V116L	-3	[47]
	A118T	+3	[47]
	Y265W	+10	[47]
RH1			
	D83N	-6	[47]
	N83D	+2	[47]
	G90S	-13	[47]
	E113D	+7	[47]
	T118A	-16	[47]
	E122Q	-20	[47]
	Q122E	+10	[47]
	I133F	blue-shift	[31]
	A164S	+2	[47]
	F261Y	+10	[47]
	Y261F	-8	[47]
	W265Y	-15	[47]
	A269T	+14	[47]
	A292S	-10	[47]
	S292A	+8	[47]
	Q122E & S292A	+26	[31]

References

46 Palczewski, K. *et al.* Crystal structure of rhodopsin: AG protein-coupled receptor. *Science* **289**, 739-745 (2000).

47 Yokoyama, S. Evolution of dim-light and color vision pigments. *Annu Rev Genom Hum Genet* **9**, 259-282 (2008).

48 Yokoyama, S. & Tada, T. The spectral tuning in the short wavelength-sensitive type 2 pigments. *Gene* **306**, 91-98 (2003).

49 Myers, P. *et al.* The Animal Diversity Web(online). Accessed at http://animaldiversity.org (2016).

50 Fricke, H., Reinicke, O., Hofer, H. & Nachtigall, W. Locomotion of the coelacanth *Latimeria chalumnae* in its natural environment. *Nature* **329**, 331-333 (1987).

51 Vega-Zuniga, T. *et al.* Does nocturnality drive binocular vision? Octodontine rodents as a case study. *PloS one* **8**, e84199 (2013).

52 Nagai, K. & Oishi, T. Behavioral rhythms of the Japanese newts, *Cynops pyrrhogaster*, under a semi-natural condition. *Int J Biometeorol* **41**, 105-112 (1998).

53 Tawa, Y., Jono, T. & Numata, H. Circadian and temperature control of activity in Schlegel's Japanese Gecko, *Gekko japonicus* (Reptilia: Squamata: Gekkonidae). *Current Herpetol* **33**, 121-128 (2014).

54 Macey, J. & Papenfuss, T. in *Natural History of the White-Inyo Range, Eastern California* (ed Clarence A Hall) 291-360 (University of California Press, 1991).

55 Stuart, B. *et al. Python bivittatus*. *The IUCN Red List of Threatened Species 2012*, e.T193451A2237271 (2012).

56 Davies, W. L. *et al.* Shedding light on serpent sight: the visual pigments of henophidian snakes. *J Neurosci* **29**, 7519-7525 (2009).

57 Jessop, T. S., Limpus, C. J. & Whittier, J. M. Nocturnal activity in the green sea turtle alters daily profiles of melatonin and corticosterone. *Horm Behav* **41**, 357-365 (2002).

58 Watanabe, Y. Y., Reyier, E. A., Lowers, R. H., Imhoff, J. L. & Papastamatiou, Y. P. Behavior of American alligators monitored by multi-sensor data loggers. *Aquat Biol* **18**, 1-8 (2013).

59 Grassman Jr, L. I., Haines, A. M., Tewes, M. E. & Silvy, N. J. Stouffer® wildlife timers as an index of vertebrate activity periods in a tropical forest. *Wildl Soc Bull* **33**, 1174-1177 (2005).

60 Bennie, J. J., Duffy, J. P., Inger, R. & Gaston, K. J. Biogeography of time partitioning in mammals. *Proc Natl Acad Sci USA* **111**, 13727-13732 (2014).
